# Non-alcoholic Fatty Liver Disease and Its Association With Left Ventricular Diastolic Dysfunction: A Systematic Review

**DOI:** 10.7759/cureus.43013

**Published:** 2023-08-06

**Authors:** Namra V Gohil, Nida Tanveer, Vijaya Krishna Makkena, Arturo P Jaramillo, Babatope L Awosusi, Javaria Ayyub, Karan Nareshbhai Dabhi, Tuheen Sankar Nath

**Affiliations:** 1 Internal Medicine, Medical College Baroda, Vadodara, IND; 2 Internal Medicine, California Institute of Behavioral Neurosciences & Psychology, Fairfield, USA; 3 Internal Medicine, University of Louisville, Louisville, USA; 4 Medicine, Osmania Medical College, Hyderabad, IND; 5 General Practice, California Institute of Behavioral Neurosciences & Psychology, Fairfield, USA; 6 Pathology and Laboratory Medicine, California Institute of Behavioral Neurosciences & Psychology, Fairfield, USA; 7 Surgical Oncology, California Institute of Behavioral Neurosciences & Psychology, Fairfield, USA

**Keywords:** a systematic review, 2d echocardiography, left ventricular diastolic dysfunction, cardiology imaging, adult cardiology, liver steatosis, non-alcoholic steatohepatitis (nash), nonalcoholic fatty liver disease (nafld), general gastroenterology, adult gastroenterology

## Abstract

The commonest cause of hepatic illness globally is non-alcoholic fatty liver disease (NAFLD). This multisystemic disease affects extrahepatic organs, including the heart. It causes cardiac remodeling and a disruption of the systolic and diastolic functioning of the left ventricle. Numerous studies have investigated the connection between NAFLD and left ventricular diastolic dysfunction (LVDD). The results, nevertheless, are often contradictory. This systematic review looked at the relationship between NAFLD and LVDD generally and among different patient groups since it is a topic of interest. A thorough search approach was used to locate relevant publications published between 2003 and 2023 using major medical databases. Studies were chosen based on the pre-established eligibility criteria; the studies selected then underwent a critical evaluation using standardized quality assessment tools. For the systematic review, 13 articles were chosen, comprising nine cross-sectional studies, three narrative reviews, and one meta-analysis. There were a total of 13,341 NAFLD patients in these studies. Data extraction and qualitative synthesis from the selected research articles were conducted to determine the relationship between NAFLD and LVDD in various patient categories. We found a significant association between NAFLD and LVDD. Therefore, patients with NAFLD should be treated early to avoid complications since they are more likely to develop cardiac dysfunction in the future.

## Introduction and background

In the United States (US) and Europe, non-alcoholic fatty liver disease (NAFLD), which affects up to 25% of the population, has epidemic proportions and is projected to rise sharply over the next 10 years [1]. In 2016, 64 million people in the US were predicted to have NAFLD, which resulted in yearly healthcare expenses of over $103 billion. Patients in the age group of 45-65 years accounted for most NAFLD expenses, and this working-age demographic had a much more significant economic burden of cardiovascular disease (CVD) [2,3].

NAFLD is a group of liver disorders that affect people who drink little to no alcohol and have at least 5% of their liver cells steatosed [4]. Non-alcoholic steatohepatitis (NASH) is the progressive and more severe form of NAFLD. Steatosis, lobular inflammation, and ballooning of hepatocytes are histological features of NASH; fibrosis is often seen too. NASH may advance to cirrhosis, a disease in which hepatocytes are replaced by scar tissue comprised of type I collagen produced by astrocytes to heal injured cells. Cirrhosis is a progressive liver condition that might cause hepatocellular cancer or need a liver transplant [5-7].

According to available data, NAFLD affects several extrahepatic organ systems, including the heart and vascular systems [8]. Coronary artery disease (CAD) is the commonest cause of morbidity and mortality in NAFLD [9]. In the US, NAFLD ranks third among the causes of liver transplantation and will probably surpass all other causes in the following years [1]. 

The prevalence and severity of NAFLD are highly correlated with various subclinical atherosclerosis indicators, including increased arterial stiffness, impaired endothelial function, increased carotid artery wall thickness, and a greater incidence of carotid atherosclerotic plaque [10-12]. Increased cardiac fatty acid oxidation is caused by the build-up of circulating free fatty acids and triglycerides in NAFLD patients' hepatic and myocardial cells. Cellular dysfunction and gene expression alterations that result in myocyte damage and cell death in an insulin-resistant condition, such as in NAFLD, lead to cardiac remodeling. NAFLD-induced inflammation and oxidative stress may also lead to insulin resistance and cardiac fibrosis, which may alter the heart’s structure [13-15].

The evidence supporting a significant connection between NAFLD and cardiac dysfunction, mainly left ventricular diastolic dysfunction (LVDD), has grown in recent years. There is some information available on the correlation between NAFLD and LVDD. However, diverse relationships between NAFLD and LVDD have been seen in studies including patients from different patient categories, including those with and without diabetes, obesity, hypertension, and variable degrees of steatosis. Tissue Doppler imaging (TDI) and two-dimensional echocardiography (2D Echo) are used to assess LVDD. Even though links between NAFLD and LVDD have been documented by several studies, some results are inconsistent, perhaps due to small sample sizes or restricted populations, or an insufficient correction for possible confounding factors. As a result, this systematic review aims to examine the existing literature and comprehend how NAFLD and LVDD are related in various patient groups.

## Review

Methods

We structured our systematic review, conducted it, and reported the results according to the Preferred Reporting Items for Systematic Reviews and Meta-analyses (PRISMA) principles and guidelines [16].

Search Strategy

An extensive search approach was used on April 17, 2023, to find pertinent publications from PubMed, PubMed Central (PMC), MEDLINE, Google Scholar, ResearchGate, and Directory of Open Access Journals (DOAJ) databases. Medical Subject Heading (MeSH) terms and appropriate keywords were used for the literature search. The MeSH terms and keywords were combined using Boolean terms such as “AND”, “OR”, and “NOT”. 

The final search strategy for PubMed, PMC, and MEDLINE was: Non-alcoholic fatty liver disease OR Non-alcoholic steatohepatitis OR Metabolic dysfunction associated fatty liver disease (MAFLD) OR NAFLD OR NASH OR MAFLD OR ( "Non-alcoholic Fatty Liver Disease/complications"[Majr] OR "Non-alcoholic Fatty Liver Disease/epidemiology"[Majr] OR "Non-alcoholic Fatty Liver Disease/pathology"[Majr] OR "Non-alcoholic Fatty Liver Disease/physiopathology"[Majr] ) AND Left ventricular diastolic dysfunction.

The keywords used for search and combined using booleans in Google Scholar, ResearchGate, and DOAJ were “Non-alcoholic fatty liver disease”, “Non-alcoholic steatohepatitis”, “Metabolic dysfunction associated fatty liver disease”, “NAFLD”, “NASH”, and “MAFLD”. Pertinent additional articles on the topic were searched from the bibliography of the selected articles.

Inclusion and Exclusion Criteria

To assess each article's eligibility, two authors independently evaluated it. Any discrepancies were then cleared up with input from the other authors. We included randomized controlled trials (RCTs), observational studies, and review articles that were pertinent to the research question and that focused on adults over the age of 18 years. Excluded from the study were case reports, letters to the editor, expert opinions, unpublished or grey literature, and articles targeting people under the age of 18.

Table 1 provides a comprehensive summary of the inclusion and exclusion criteria.

**Table 1:** Inclusion and Exclusion Criteria RCTs, Randomized controlled trials; NAFLD, Non-alcoholic fatty liver disease

Inclusion Criteria	Exclusion Criteria
1. Articles published between 2003 and 2023	1. Articles published before 2003
2. Articles in English	2. Articles in languages other than English
3. Articles focusing on the population above 18 years of age	3. Articles focusing on the population under the age of 18 years
4. Observational studies, RCTs, and review articles (Narrative Review and Systematic Review)	4. Case reports, letters to the editor, expert opinions, non-human studies, unpublished articles
5. Articles on NAFLD	5. Articles on alcoholic, infective, drug-induced, autoimmune, and infiltrative liver disease, for example, hemochromatosis and Wilson’s disease
6. Full-text articles	6. Articles only containing abstract
7. Articles relevant to the research topic	7. Articles not relevant to the research topic

Quality Appraisal of Studies/Bias

**Figure 1:**
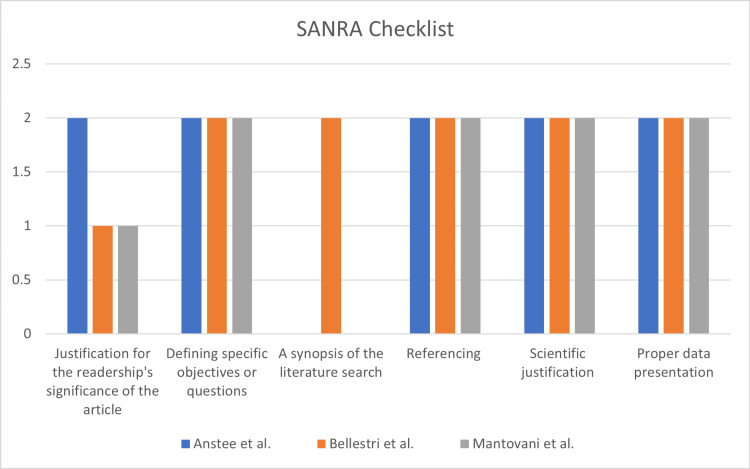
SANRA Checklist for Narrative Reviews SANRA, Scale for the Assessment of Narrative Review Articles Anstee et al. [17], Bellestri et al. [18], Mantovani et al. [19]

**Figure 2:**
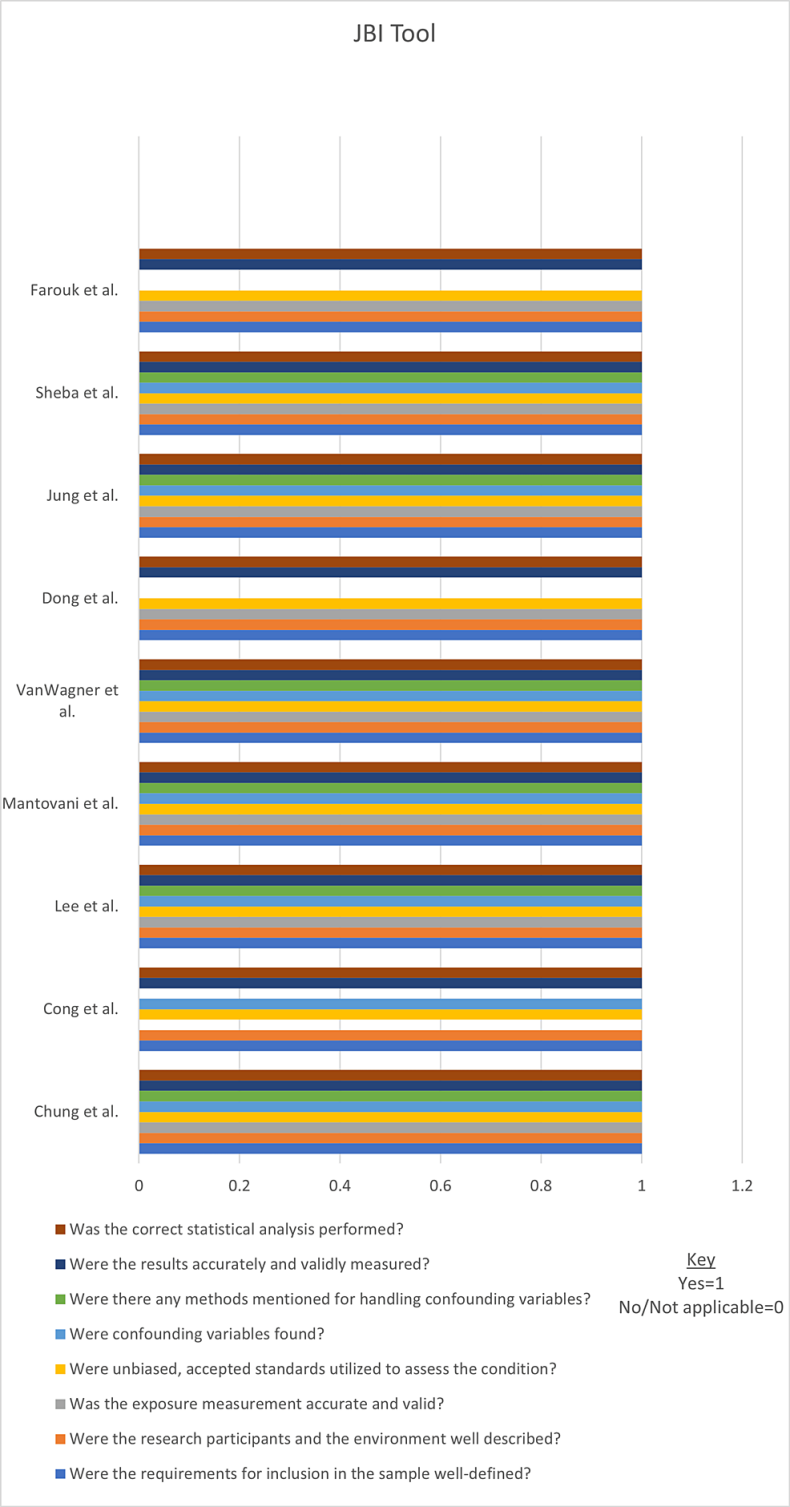
JBI Tool JBI, Joanna Briggs Institute Chung et al. [20], Cong et al. [21], Lee et al. [22], Mantovani et al. [23], VanWagner et al. [24], Dong et al. [25], Jung et al. [26], Sheba et al. [27], Farouk et al. [28]

We evaluated the 13 chosen articles' quality and risk of bias using established quality evaluation tools. All articles met the medium/high-quality criteria and were included in our systematic review. For the critical evaluation of the studies, the following quality assessment tools were utilized: (i) the Scale for the Assessment of Narrative Review Articles (SANRA) checklist, for traditional/narrative reviews; (ii) the Joanna Briggs Institute (JBI) critical appraisal checklist, for cross-sectional studies; and (iii) the Assessment of Multiple Systematic Reviews (AMSTAR) tool for systematic reviews and meta-analyses. Tables 2-4, and Figures 1-2 include each article's comprehensive quality analysis and overall scores.

**Table 2:** Summary of the SANRA Checklist SANRA, Scale for the Assessment of Narrative Review Articles

	Anstee et al. [17]	Bellestri et al. [18]	Mantovani et al. [19]
Justification for the readership's significance of the article	The significance is clearly justified-2	The significance is indicated but not clearly justified-1	The significance is indicated but not clearly justified-1
Defining specific objectives or questions	One or more specific objectives are established-2	One or more specific objectives are established-2	One or more specific objectives are established-2
A synopsis of the literature search	The search methodology is not disclosed-0	Details of the literature search are provided, including the search keywords and inclusion criteria-2	The search methodology is not disclosed-0
Referencing	References are used to support critical statements-2	References are used to support critical statements-2	References are used to support critical statements-2
Scientific justification	Overall, relevant evidence is available-2	Overall, relevant evidence is available-2	Overall, relevant evidence is available-2
Proper data presentation	In general, adequate presentation of relevant outcome data-2	In general, adequate presentation of relevant outcome data-2	In general, adequate presentation of relevant outcome data-2
Total Score	10/12	11/12	9/12
Quality	High	High	Medium/High

**Table 3:** JBI Tool for Quality Appraisal of Cross-sectional Studies JBI, Joanna Briggs Institute; Y, Yes; N, No; NA, Not applicable

Item	Chung et al. [20]	Cong et al. [21]	Lee et al. [22]	Mantovani et al. [23]	VanWagner et al. [24]	Dong et al. [25]	Jung et al. [26]	Sheba et al. [27]	Farouk et al. [28]
Were the requirements for inclusion in the sample well-defined?	Y	Y	Y	Y	Y	Y	Y	Y	Y
Were the research participants and the environment well described?	Y	Y	Y	Y	Y	Y	Y	Y	Y
Was the exposure measurement accurate and valid?	Y	NA	Y	Y	Y	Y	Y	Y	Y
Were unbiased, accepted standards utilized to assess the condition?	Y	Y	Y	Y	Y	Y	Y	Y	Y
Were confounding variables found?	Y	Y	Y	Y	Y	NA	Y	Y	NA
Were there any methods mentioned for handling confounding variables?	Y	N	Y	Y	Y	NA	Y	Y	NA
Were the results accurately and validly measured?	Y	Y	Y	Y	Y	Y	Y	Y	Y
Was the correct statistical analysis performed?	Y	Y	Y	Y	Y	Y	Y	Y	Y
Total Score	8/8	6/8	8/8	8/8	8/8	6/8	8/8	8/8	6/8
Quality	High	Medium/High	High	High	High	Medium/High	High	High	Medium/High

**Table 4:** AMSTAR Checklist for Quality Appraisal of Systematic Reviews AMSTAR, Assessment of Multiple Systematic Reviews; Y, Yes; N, No; RoB, Risk of bias; PICO, Population/Intervention/Comparison/Outcome

Item	Wang et al. [29]
Did the PICO components meet the review's inclusion criteria and research questions?	Y
Did the report explicitly mention that the review process had been created before the review's conduct, and did the information justify any substantial divergences from the protocol?	Y
Did the review authors explain why they chose to include the research designs?	Y
Did the review authors do a thorough literature search?	Partial Yes
Was the study selection process carried out in duplicate by the review authors?	Y
Did the authors of the review extract data in duplicate?	Y
Did the review authors mention the papers they omitted and explain why?	Partial Yes
Did the review authors sufficiently describe the studies that were included?	Y
Did the review authors use an adequate approach to determine the risk of bias (RoB) in each study?	N
Have the review's authors provided information about the funding sources for the research they reviewed?	Y
Did the review authors use the proper techniques for the statistical combination of results if a meta-analysis was conducted?	Y
If a meta-analysis was conducted, did the review authors consider how the outcomes of individual studies' risk of bias would affect the findings of the meta-analysis or other evidence synthesis?	N
When interpreting or presenting the review results, did the authors consider RoB in the individual studies?	N
Did the review's authors adequately explain and address any heterogeneity in the study's results?	Y
Did the review authors adequately investigate publication bias (small study bias) if they used quantitative synthesis, and how would it have affected the review's results?	Y
Did the review’s authors disclose any possible conflicts of interest, including any funds they may have received for conducting it?	Y
Total Score and Quality	12/16 (Medium/High Quality)

Data Extraction

Two authors independently extracted the data from the selected articles. The articles were examined for (i) type of study, (ii) the number of patients with NAFLD, (iii) investigation modality used for diagnosis of NAFLD, (iv) parameters for LVDD studied, and (v) study outcome.

Results

**Figure 3:**
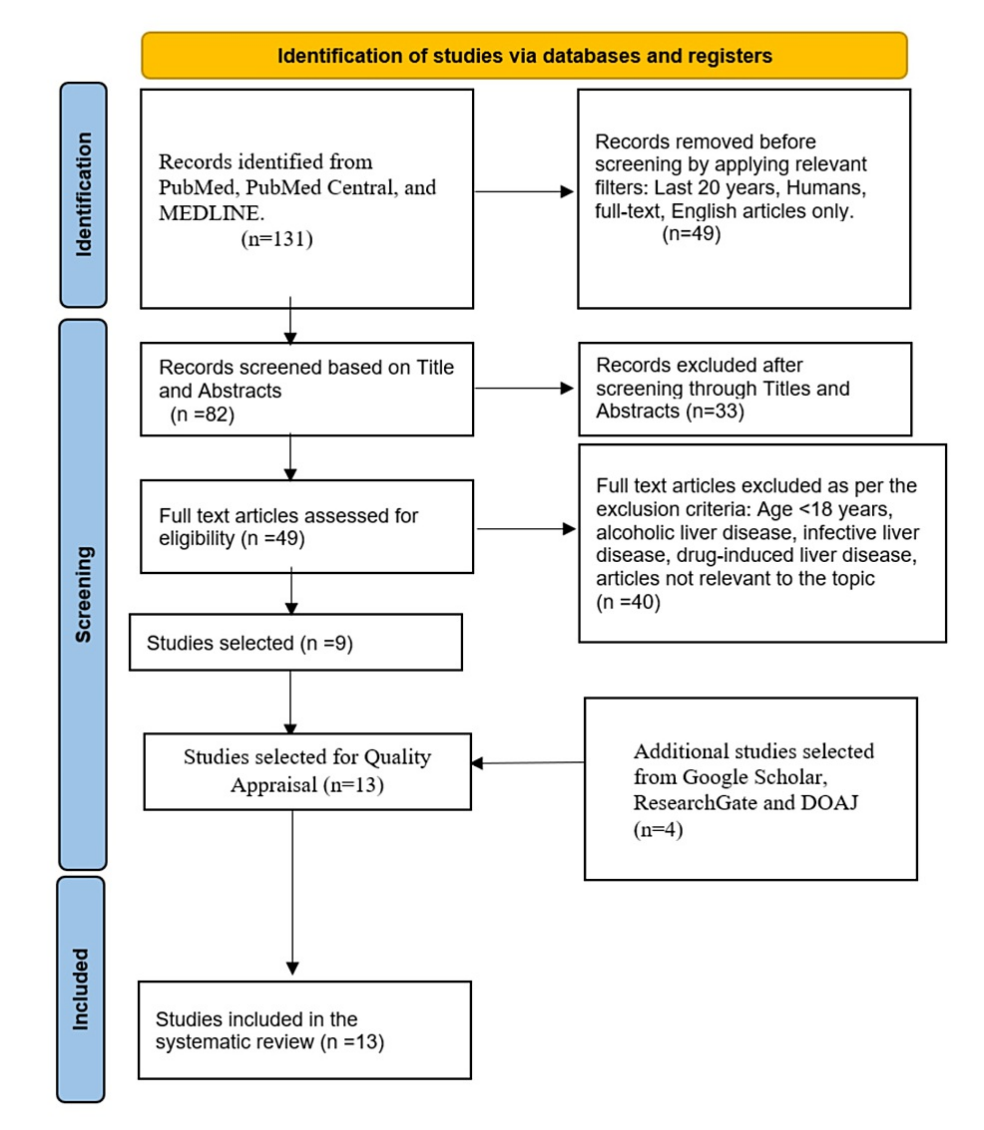
PRISMA Flow Diagram PRISMA, Preferred Reporting Items for Systematic Reviews and Meta-Analyses

In the first MEDLINE, PubMed, and PMC database searches, 131 publications were found. Of these, 49 were removed after applying the proper filters based on our eligibility criteria. Two authors evaluated the remaining publications (n = 82) using the titles, abstracts, and predetermined inclusion and exclusion criteria. After the meticulous screening, nine articles that dealt with our research topic remained. Four more publications were found by looking for our topic's relevant keywords in Google Scholar, ResearchGate, and DOAJ. The 13 articles at the end of this were subjected to a thorough quality/bias evaluation utilizing established quality assessment techniques. Our systematic review included all 13 of the final studies. Figure 3 shows the PRISMA flow diagram.

Of the 13 articles included, nine were cross-sectional studies, three were narrative reviews, and one was a systematic review with meta-analysis. Many of the studies were common in the review articles. After removing the duplicates, we included 13,341 patients with NAFLD from the 13 articles investigating the association between NAFLD and LVDD. We separated our studies into several sub-groups, such as those looking at the relationship between NAFLD and LVDD in patients with type 2 diabetes mellitus (DM) and non-obese people, and those investigating the relationship between NAFLD and LVDD in general.

After analysis, we found a significant association between NAFLD and the risk of developing LVDD. This association became stronger as the degree of NAFLD increased. In one sub-group, we investigated the relationship between NAFLD and LVDD in diabetic patients. We found that the association is more significant in people with diabetes than non-diabetics. In another sub-group, we found that the association between NAFLD and LVDD is more significant in non-obese than obese individuals.

Discussion

We qualitatively synthesized data from the 13 studies we chose for our systematic review and discovered that patients with NAFLD are more likely to develop LVDD. These studies studied several echocardiography parameters related to LVDD and employed various investigations to diagnose NAFLD. So far as we know, there hasn't been a systematic review study that looks at the relationship between NAFLD and LVDD in general and in specific patient groups, like NAFLD patients with type 2 DM or NAFLD patients who aren't obese.

The gold standard for diagnosing NAFLD is liver biopsy; however, it is an invasive technique, so ultrasonography (USG) is more commonly used for diagnosing NAFLD. The characteristic features of NAFLD on USG are liver parenchymal brightness, the contrast between the brightness of liver and renal parenchyma, focal sparing of liver parenchyma, bright walls of hepatic vessels, and impaired visualization of intrahepatic borders and the diaphragm [30-32].

The echocardiography parameters used for the assessment of LVDD are the peak velocities of the early (E) and late (A) phases of mitral inflow during diastole, deceleration time (DT) of E velocity, E/A ratio, early diastolic (e’) and late diastolic (a’) velocities of the septal and lateral mitral valve annulus; E/e’ ratio which indicates left ventricular filling pressure; left ventricular mass index (LVMI) and left atrial maximum volume index (LAVImax). Pulse wave Doppler measures E and A velocities, whereas e’ and a’ velocities are measured by tissue Doppler imaging (TDI) [33,34]. The echocardiographic parameters used as the markers of LVDD are: higher A velocity, lower E/A ratio, decreased e’ velocity, increased E/e’ ratio, greater LVMI, and greater LAVImax [35].

Association Between NAFLD and LVDD in General Population

Six of our selected studies tried to find the association between NAFLD and LVDD in the general population while not dividing the chosen sample into subgroups like diabetics and non-obese. This included three narrative/traditional reviews and three cross-sectional studies. These studies varied in their study design, eligibility requirements, sample size, demographic and geographic characteristics, and outcomes.

In their narrative review, Anstee et al. tried to find the risk of LVDD, cardiomyopathy, and cardiac arrhythmias in patients with NAFLD [17]. The review included 22 articles assessing the risk of LVDD. These 22 articles studied a total of 9311 patients with NAFLD. This study found a significant correlation between the severity of NAFLD and the likelihood of LVDD, cardiomyopathy, and arrhythmias (mainly atrial fibrillation).

Ballestri et al., in their narrative review, assessed the risk of LVDD and arrhythmic complications in patients with NAFLD [18]. They included six articles with 233 adult NAFLD patients to find the risk of developing LVDD in them. According to the review, there is a clear association between NAFLD and an increased risk of LVDD and arrhythmogenic consequences.

In their narrative review, Mantovani et al. summarized the literature on the association between NAFLD and LVDD, CHD, and arrhythmias [19]. To find the association between NAFLD and LVDD in adults, they included 15 articles with 1499 NAFLD patients. Their review found a strong association between NAFLD, LVDD, cardiac arrhythmias, and CHD.

In a population-based cross-sectional study, VanWagner et al. tried to find the association between NAFLD and LVDD by drawing their sample from participants in the Coronary Artery Risk Development in Young Adults (CARDIA) cohort study's 25-year follow-up assessment [24,36]. A total of 271 patients were diagnosed with NAFLD using computed tomography (CT) in the study conducted by VanWagner et al., and NAFLD was associated with markers of diastolic dysfunction such as lower e’ velocity, lower E/A ratio, and increased E/e’ ratio after adjusting for BMI, health behaviors, and demography. After adjustment for visceral adipose tissue (VAT), only e’ velocity and E/e’ ratio remained significant, whereas the association with only e’ velocity remained significant after adjustment for markers of adiposity [24]. The major limitation of the study by VanWagner et al. was the use of a CT scan, which is relatively less sensitive for diagnosing NAFLD. Therefore, this may have introduced bias in the results. In their study, Kodama et al. determined that the best way to predict the amount of pathologic fat in the liver is to analyze its attenuation on unenhanced CT scans [37]. Park BJ et al.'s study shows that VAT is a distinctive risk factor for hepatic steatosis [38].

A large cross-sectional study including 20,821 Korean men and women was conducted by Jung et al. [26]. On USG, 6171 individuals were diagnosed with NAFLD. The study participants were divided into three subgroups: normal, mild NAFLD, and moderate to severe NAFLD. They concluded that after adjusting for various confounding factors, NAFLD is significantly associated with LVDD. The echocardiographic markers of LVDD, which showed significant association included e’ velocity, E/A ratio, E/e’ ratio, relative wall thickness (RWT) of the left ventricle, and LVMI. This association became stronger as the severity of NAFLD increased. The strength of this study was the large sample size. However, there was selection bias since most research participants were company workers and their family members.

Farouk et al. performed a small cross-sectional study using echocardiography to evaluate LVDD in 35 patients with non-alcoholic liver cirrhosis [28]. Mitral A velocity, e’ velocity, E/A ratio, and E/e’ ratio showed significant association in patients compared to healthy controls. However, the mitral inflow parameters were normal when checked in patients with raised left ventricle filling pressure. The authors concluded that many factors other than LVDD could be responsible for these patients' normal mitral inflow parameters. These could be tense ascites, increased heart rate, anemia, decreased intravascular volume, and difficulty performing the Valsalva maneuver properly due to ascites during echocardiography in these patients.

The analysis of the above studies shows that NAFLD is significantly associated with LVDD, and the association becomes more significant as the severity of NAFLD increases. A brief description of each study is given in Table 5.

**Table 5:** Studies investigating the association between NAFLD and LVDD in General Population NAFLD, Non-alcoholic fatty liver disease; LVDD, left ventricular diastolic dysfunction; CHD, coronary heart disease; LV; left ventricle; RWT, relative wall thickness; VAT, visceral adipose tissue; USG, ultrasonography

Author and publication year	Study Design	Number of participants with NAFLD	The investigation used for the diagnosis of NAFLD	Results
Anstee et al., 2018 [17]	Narrative review	9311	Liver USG	NAFLD was strongly associated with LVDD, cardiomyopathy, and arrhythmias
Ballestri et al., 2014 [18]	Narrative review	233	Liver USG	NAFLD was associated with LVDD and arrhythmias
Mantovani et al., 2016 [19]	Narrative review	1499	Liver USG	NAFLD was associated with LVDD, CHD, and arrhythmias
VanWagner et al., 2015 [24]	Cross-sectional study	271	CT	NAFLD was shown to be substantially linked to e', E/A, and E/e'. E/e' and e' remained significant after adjusting for VAT. However, only e' remained significant after adjusting for adiposity markers.
Jung et al., 2016 [26]	Cross-sectional study	6171	Liver USG	E/A, e’, E/e’, LV RWT, and LVMI were associated with NAFLD
Farouk et al., 2017 [28]	Cross-sectional study	35	Liver USG	A, E/A, e’, and E/e’ were associated with non-alcoholic cirrhosis patients. However, the mitral inflow parameters were relatively normal in patients with elevated LV filling pressure.

Association Between NAFLD and LVDD in Individuals With Type 2 DM

Five of the 13 included articles examined the relationship between NAFLD and LVDD in people with type 2 DM. Four of these five studies were cross-sectional studies, and one was a systematic review with meta-analysis. These studies differed in the type of study design, eligibility criteria, sample size, demographic and geographic parameters, and their results.

A cross-sectional study on 222 people with type 2 DM who had neither heart nor liver problems was carried out by Mantovani et al. [23]. Among them, 66 were female, and 156 were male. On USG, NAFLD was identified in 158 of the 222 individuals. Compared to type 2 DM patients without NAFLD, LVDD was substantially more common in NAFLD patients with type 2 DM. Even after controlling for confounding variables, the association remained significant. Lower e' velocity, greater E/e' ratio, and higher left ventricular end-diastolic pressure were the LVDD echocardiographic characteristics that were more significant in patients with NAFLD. However, the outcomes of this research could not be extrapolated to other diabetic groups since it only comprised type 2 DM patients attending an outpatient clinic.

Dong et al. classified 97 type 2 DM patients into three groups in a cross-sectional study based on USG results: Group A consisted of 30 people who did not have NAFLD, Group B of 32 people who had mild NAFLD, and Group C of 35 people who had moderate-to-severe NAFLD [25]. In addition to traditional echocardiographic measures, three-dimensional speckle-tracking echocardiography (3D-STE), a reliable approach for assessing LV function, was utilized in this study [39]. The left ventricular global radial, longitudinal, circumferential, and area strains were among the 3D-STE parameters. Compared to groups B and A, the study’s findings indicated that group C had lower left ventricle strains, lower e' velocity, higher E/e’ ratio, and higher LAVImax. The study also showed that the glycated hemoglobin (HbA1c) negatively impacted all strains of 3D-STE. The study's principal flaws were the small sample size and lack of adjustment for confounding variables.

Lee et al. conducted a cross-sectional study on 606 type 2 DM individuals [22]. Of the 606 patients, 355 were diagnosed with NAFLD. The subjects with NAFLD were divided into subgroups: NAFLD patients with simple steatosis and NAFLD patients with advanced liver fibrosis using NAFLD fibrosis score (NFS) [40]. The NAFLD group had a substantially higher prevalence of LV diastolic dysfunction than the non-NAFLD group. Even after controlling for confounding variables such as age, sex, BMI, hypertension, diabetes duration, and HbA1c, the association remained significant. Patients with advanced hepatic fibrosis showed greater diastolic dysfunction than patients with simple steatosis. After accounting for insulin resistance and cardiometabolic risk variables, liver fibrosis was shown to be independently linked with diastolic dysfunction in multivariable logistic regression. The association remained significant only in people without insulin resistance [22].

Sheba et al. carried out a cross-sectional study in which 40 type 2 DM participants were separated into two groups of 20 each, depending on the presence or absence of NAFLD [27]. Based on the hepato-renal index (HRI), individuals with NAFLD were classified as having mild, moderate, or severe steatosis [41]. In comparison to patients with type 2 DM alone, those with type 2 DM and NAFLD had higher peak A, higher left atrial volume, higher peak E/A, lower peak e', and higher left ventricle filling pressure index (E/e'). They discovered a statistically significant difference between the various degrees of steatosis determined by HRI in terms of the peak e' and E/e'. Peak e' and the E/e' increased with increasing steatosis (i.e., more LVDD). Additionally, the multivariate analysis revealed that the only independent variable linked with LVDD in patients with type 2 DM and NAFLD is HRI. The study's shortcomings were a limited sample size, greater e' velocity, and lower E/A ratio as the degree of steatosis increased, all contradicting the prevailing consensus.

Wang et al. conducted a meta-analysis to investigate whether NAFLD is a sign of LVDD in type 2 DM patients [29]. The meta-analysis comprised 10 cross-sectional studies with 1800 type 2 DM patients, of which 1124 had NAFLD. The study's findings revealed that the NAFLD group had decreased E/A ratio, increased A velocity, decreased e', greater E/e' ratio, higher LAVImax, and higher LVMI than patients without NAFLD, all of which pointed to a greater risk of LVDD. The study's main limitation was the heterogeneity of several echocardiographic parameters, due to which the random-effects model was used, producing less reliable outcomes than the fixed-effects model.

The analysis of the above studies shows that NAFLD in type 2 DM patients is significantly associated with LVDD, and the association becomes more vital as the degree of steatosis and fibrosis increases. A brief description of each study is given in Table 6.

**Table 6:** Studies investigating the association between NAFLD and LVDD in Type 2 DM individuals NAFLD, Non-alcoholic fatty liver disease; LVDD, left ventricular diastolic dysfunction; LV, left ventricle; USG, ultrasonography; LVMI, left ventricular mass index; LAVImax, left atrial maximum volume index

Author and publication year	Study Design	Number of individuals with NAFLD	The investigation used for the diagnosis of NAFLD	Results
Mantovani et al., 2015 [23]	Cross-sectional	158	Liver USG	The LVDD echocardiographic parameters more significant in individuals with NAFLD were decreased e' velocity, an increased E/e' ratio, and an increased LV end-diastolic pressure.
Dong et al., 2019 [25]	Cross-sectional	67	Liver USG	Patients with moderate to severe NAFLD showed lower global radial, circumferential, area, and longitudinal strains; lower septal and lateral e' velocities, greater E/e' ratios, and higher LAVImax values.
Lee et al., 2019 [22]	Cross-sectional	355	Liver USG	NAFLD was significantly associated with LVDD only in patients without insulin resistance
Sheba et al., 2022 [27]	Cross-sectional	20	Liver USG	Type 2 DM and NAFLD patients had higher peak late diastolic flow velocities (A), higher left atrial volume, higher peak early/late diastolic flow velocities (E/A), higher peak early diastolic annular velocities (e'), and higher left ventricle filling pressure index (E/e') compared to patients with type 2 DM alone.
Wang et al., 2023 [29]	Systematic Review and Meta-analysis	1124	Liver USG in 8 studies and Liver transient elastography in 2 studies	The research showed that patients with NAFLD had considerably decreased E/A ratio, increased A velocity, decreased e, increased E/e' ratio, increased LAVImax, and increased LVMI than those without NAFLD.

Association Between NAFLD and LVDD in the Non-obese Population

Two of our selected studies investigated the association between NAFLD and LVDD in non-obese adults. Both of these are cross-sectional studies.

Chung et al. conducted a cross-sectional study on 3300 subjects, of which 2582 were diagnosed with NAFLD on USG [20]. The study was published in 2018. The NFS was calculated, and patients were classified into three categories based on lack of fibrosis, NAFLD without advanced fibrosis, and NAFLD with advanced fibrosis. Compared to controls, patients with NAFLD had a higher E/e’ ratio, lower e’, and an increased left atrial diameter. This association remained significant even after adjusting for confounders. When the data were stratified according to BMI and after adjusting for other risk factors of LVDD, the study found that the increased association between NAFLD fibrosis grade and LVDD was only significant in non-obese patients. This study, however, had a misclassification bias as it was a retrospective study and might have a limited assessment of left ventricular diastolic function.

A cross-sectional study on 316 non-obese patients was conducted by Cong et al. [21]. The study was published in March 2023. Of 316 patients, 72 were diagnosed with NAFLD on USG. Non-obese NAFLD participants had a decreased E/A ratio than controls, and the prevalence of the E/A ratio <1 was more in the NAFLD group. The main limitation of this study was the use of only E and E/A values for the diagnosis of NAFLD, which are less sensitive and specific than other echocardiographic parameters. It is challenging to diagnose patients with grade 2 diastolic dysfunction with only an E/A ratio, as this ratio is mostly in the normal range in these patients.

The analysis of the above two studies shows that the association between increased severity of NAFLD and LVDD is more significant in non-obese adults than obese adults.

Possible Pathophysiology Responsible for Cardiac Complications in Patients With NAFLD

The exact pathophysiological mechanisms responsible for developing cardiac complications in NAFLD are still uncertain. NAFLD is characterized by altered gut microbiota (dysbiosis) and low-grade systemic inflammation in adipose tissue. This causes the release of various oxidative metabolites, pro-inflammatory cytokines (interleukin-1, interleukin-6, tumor necrosis factor-alpha, c-reactive protein), and vasoactive and thrombogenic mediators (transforming growth factor-beta, fibrinogen, plasminogen activator inhibitor-1) which cause myocardial remodeling, ultimately leading to major cardiac complications, including diastolic dysfunction [17].

Potential Treatment Options

There is no authorized medication to treat NAFLD/NASH. However, drugs that address the pathophysiology of NAFLD may be used, which may indirectly help lower insulin resistance and extrahepatic complications. Lifestyle modifications are the mainstay of treatment for NAFLD [42]. This includes a low-calorie diet and physical exercise. Statins combined with ezetimibe can be helpful by reducing atherogenesis [43]. Angiotensin-receptor blockers are beneficial by lowering blood pressure and improving insulin resistance [44]. Glucagon-like peptide-1 analog like exenatide helps by suppressing appetite and causing weight loss [45].

Limitations

This systematic review has certain limitations. First, most of the articles included are cross-sectional studies. As a result, it is highly challenging to investigate the temporal association and causal link between NAFLD and LVDD. Second, rather than using liver biopsy and histology, the gold standard for identifying NAFLD, the majority of the articles reviewed employed USG. Because USG might miss a diagnosis of steatosis when the hepatic fat content is less than 30%, there was selection bias. Third, the criteria for diagnosing the degree of diastolic dysfunction have changed several times in the last two decades with diminished reliance on the E/A ratio. This could have confounded the data. Fourth, Asian patients made up the bulk of the research population. Therefore, it's possible that the findings can't be applied to all ethnic groups globally. Fifth, since observational studies comprise the bulk of the research, there is an inherent selection bias. Sixth, there is a language bias since only studies in English were included, and additional studies in other languages may have shown stronger associations. Seventh, there is information bias brought on by the lack of standardization and accessibility of data gathered from numerous databases throughout the globe.

## Conclusions

This comprehensive systematic review demonstrates a strong association between the prevalence and severity of NAFLD and the risk of developing LVDD. Our research findings support the notion that NAFLD is a disease affecting numerous organ systems. However, whether NAFLD is only an association in LVDD patients due to shared metabolic risk factors or an independent risk factor for LVDD must be studied in detail, necessitating high-quality RCTs. Our review demonstrates that LVDD and heart failure are associated with NAFLD. It emphasizes the need for more studies that explore the significance of early detection and timely treatment for NAFLD patients to avoid cardiac dysfunction. 
